# Transarterial Embolization and Percutaneous Ethanol Injection as an Effective Bridge Therapy before Liver Transplantation for Hepatitis C-Related Hepatocellular Carcinoma

**DOI:** 10.1155/2016/9420274

**Published:** 2015-12-27

**Authors:** Marcio F. Chedid, Leandro A. Scaffaro, Aljamir D. Chedid, Antonio C. Maciel, Carlos Thadeu S. Cerski, Matheus J. Reis, Tomaz J. M. Grezzana-Filho, Alexandre de Araujo, Ian Leipnitz, Cleber D. P. Kruel, Mario R. Alvares-da-Silva, Cleber R. P. Kruel

**Affiliations:** ^1^Liver and Pancreas Transplant and Hepatobiliary Surgery Unit, Hospital de Clinicas de Porto Alegre, Federal University of Rio Grande do Sul (UFRGS), Sixth Floor, Room 600, Rua Ramiro Barcelos 2350, 90035-903 Porto Alegre, RS, Brazil; ^2^Interventional Radiology Unit, Hospital de Clínicas de Porto Alegre, Universidade Federal do Rio Grande do Sul (UFRGS), Porto Alegre, Brazil; ^3^Division of Pathology, Hospital de Clínicas de Porto Alegre, Universidade Federal do Rio Grande do Sul (UFRGS), Porto Alegre, Brazil; ^4^Division of Gastroenterology and Hepatology, Hospital de Clínicas de Porto Alegre, Universidade Federal do Rio Grande do Sul (UFRGS), Porto Alegre, Brazil

## Abstract

*Background*. Transarterial chemoembolization alone or in association with radiofrequency ablation is an effective bridging strategy for patients with hepatocellular carcinoma awaiting for a liver transplant. However, cost of this therapy may limit its utilization. This study was designed to evaluate the outcomes of a protocol involving transarterial embolization, percutaneous ethanol injection, or both methods for bridging hepatocellular carcinomas prior to liver transplantation. *Methods*. Retrospective review of all consecutive adult patients who underwent a first liver transplant as a treatment to hepatitis C-related hepatocellular carcinoma at our institution between 2002 and 2012. Primary endpoint was patient survival. Secondary endpoint was complete tumor necrosis. *Results*. Forty patients were analyzed, age 58 ± 7 years. There were 23 males (57.5%). Thirty-six (90%) out of the total 40 patients were within Milan criteria. Complete necrosis was achieved in 19 patients (47.5%). One-, 3-, and 5-year patient survival were, respectively, 87.5%, 75%, and 69.4%. Univariate analysis did not reveal any variable to impact on overall patient survival. *Conclusions*. Transarterial embolization, ethanol injection, or the association of both methods followed by liver transplantation comprises effective treatment strategy for hepatitis C-related hepatocellular carcinoma. This strategy should be adopted whenever transarterial chemoembolization and/or radiofrequency ablation are not available options.

## 1. Introduction

Hepatocellular carcinoma (HCC) is the sixth most common cancer worldwide and the third leading cause of cancer-related death [1]. Liver transplantation is the treatment of choice for patients with decompensate cirrhosis and early HCC as it removes both the tumor and the underlying liver disease [2]. As many patients face a long time on the waiting list, local tumor therapies have played an important role in avoiding list dropouts and improving posttransplant outcomes [3, 4].

It is still a matter of debate what would be the ideal bridging protocol for HCCs before liver transplantation. There is conflicting evidence about whether the addition of chemotherapy provides survival advantage to transarterial embolization (TAE) alone, especially as neoadjuvant therapy prior to liver transplantation [5]. Nevertheless, transarterial chemoembolization (TACE) with or without adjunctive radiofrequency ablation (RFA) remains the main bridging/downstaging strategy employed by most of the transplant centers worldwide.

Since TACE was not available at our center, TAE and percutaneous ethanol injection (PEI) were the only strategies available at our institution until recently. The aim of this study is to analyze the outcomes of a protocol based on TAE, PEI, or the association of both modalities for bridging HCCs before liver transplantation.

## 2. Methods

We performed a retrospective review of all consecutive adult patients who underwent first orthotopic whole-graft liver transplant from a deceased donor as treatment for HCC at our institution between March 2002 and March 2012. All consecutive hepatitis C virus- (HCV-) related HCC patients treated with TAE, PEI, or both bridging therapies before liver transplantation at our center were included. HCC patients without HCV infection were excluded, as well as HCC patients who were HCV positive but received other local anticancer therapies such as RFA and/or TACE at other centers. Patients whose HCC did not receive any local therapy prior to transplant also were excluded from this study.

Demographic, procedural, and follow-up data were collected and analyzed. Diagnosis of HCC was based on the results of ultrasound, computed tomography (CT), hepatic angiography, and/or magnetic resonance imaging (MRI) and followed the guidelines of the European Association for the Study of the Liver (EASL) and the American Association of the Study of Liver Diseases (AASLD) [6, 7]. Liver biopsy was performed for the rare cases where there was diagnostic uncertainty.

Bridging therapy for each HCC patient was decided by consensus of liver transplant surgeons, hepatologists, and interventional radiologists of our service. Treatment protocol was based on TAE for tumors greater than 2 cm. PEI was performed whenever technically feasible. All HCC transplanted patients were within Milan criteria at the time of transplant. Only a small subset of this cohort was composed of individuals that achieved Milan criteria only after downstaging with TAE.

TAE was performed by an interventional radiologist through a femoral access point under sedation. A Cobra of Mikaelson 5 F catheter was used to achieve selective catheterization and arteriogram of celiac trunk and superior mesenteric artery. Tumor feeding artery was selectively catheterized using a 2.8 F microcatheter (Progreat, Terumo). Polyvinyl alcohol (PVA) or microspheres with particles sized 100–300 micrometers were infused. The number of vials used for each patient was variable depending on the number and size of the nodules. Follow-up images were obtained 4–6 weeks after the procedure and the need for subsequent therapies was decided on the basis of residual vascularity. All patients listed with a known HCC had a protocol CT or MRI imaging to access tumor growth every three months until liver transplant.

PEI was performed by an interventional radiologist through CT or ultrasound puncture-guided with 20-gauge needle and under sedation. Only HCC nodules smaller than 3 cm were subjected to PEI. Ethanol was injected until the nodule was completely filled by the echogenic effect of the fluid.

Dedicated liver pathologists evaluated all liver explants and recorded the following tumor characteristics: number of nodules, size, and presence of complete necrosis (only necrotic material with no residual tumor). Each tumor nodule was sectioned, stained, and carefully examined for microvascular invasion. All lesions that had viable tumor were graded by differentiation (well, moderate, or poor).

Patients were followed up until their death or the end of the study period. None of the patients were lost to follow-up. The primary endpoint was overall patient survival. Overall survival was calculated from the date of liver transplant to the date of the death or until the last follow-up visit. Secondary endpoint was the presence of complete tumor necrosis in the explanted liver.

Continuous variables were expressed using median (range). Categorical variables were compared using chi-square test and continuous variables with Mann-Whitney *U* test or *t*-test as appropriate. For primary endpoint, variables that were statistically significant in the univariate analysis (*p* < 0.1) were pulled into multivariable models in order to identify independent risk factors associated with the two study endpoints. Survival was analyzed using Kaplan-Meier method, and comparisons were performed using log-rank test. For all analyses, a *p* value <0.1 was considered statistically significant. Analyses were performed using JMP statistical package, version 12 (Statistical Discovery, SAS, Cary, NC, USA, 2011) and SPSS 18.0 for Windows.

## 3. Results

A total of 49 patients with HCV and HCC were transplanted at our center during the study period. Two of those 49 patients were excluded because they had a mixed tumor (HCC plus cholangiocarcinoma). Another two patients were excluded because HCC was an incidental finding not detected preoperatively and diagnosed only in the explanted liver. Five additional patients were excluded from this study because they underwent TACE and/or RFA therapy for their HCC at another center before being referred to our hospital. Forty patients were analyzed in this study, being 23 males (57.5%) and 17 females (42.5%), mean age of 58 ± 7 years (range 38–71). Thirty-six (90%) out of the total 40 patients fulfilled Milan criteria. The remaining 4 patients were listed based on up-to-seven criteria and were transplanted within Milan criteria after downstaging.

The median calculated MELD score for this cohort at the time of transplantation was 13 (range 6–22). We performed an analysis comparing the two subgroups of patients (MELD score 15 and above versus lower than 15). A MELD score 15 or higher was not associated with a lower overall survival (*p* = 0.135). Median wait-list time was approximately 7 months. According to pretransplant imaging studies, 23 (57.5%) out of the total 40 patients had a single HCC tumor.

Nineteen out of the total 40 patients underwent TAE bridging/downstaging procedures with or without association to PEI ablation. The remaining 21 patients had their HCC treated only by PEI procedures. The mean time between TAE and liver transplant date was 205 days (range 6–495 days). The median number of TAE sessions was 1, and the maximum was 3. The median number of PEI sessions was 1 (range 0–7 sessions). Complete necrosis was identified in the explants of 19 patients (47.5%). Major toxicity occurred in only one out of total 34 TAE procedures (one patient developed a liver abscess, successfully treated without the need for surgery).

**Figure 1:**
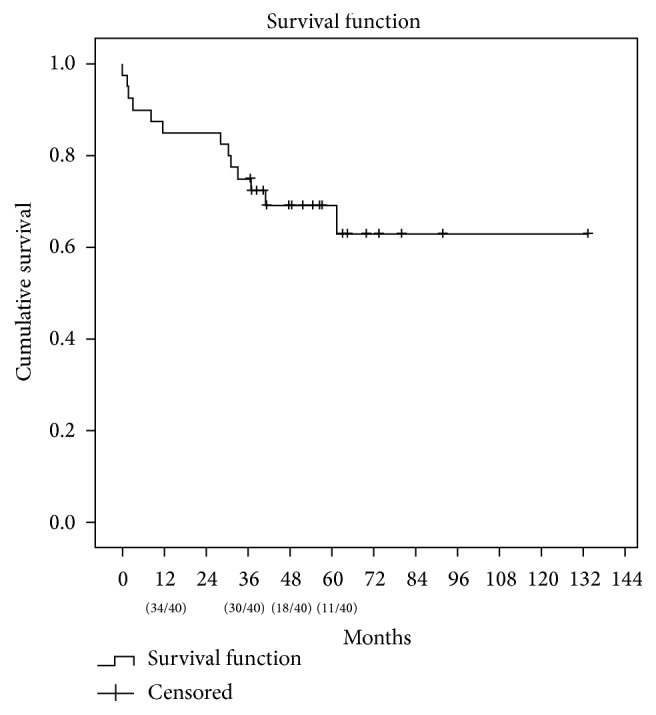
Patient survival (*n* = 40).

Median follow-up was 1520 days. There were overall 13 (32.5%) deaths, 4 of those occurring on the first 90 posttransplant days. One-year, 3-year, and 5-year patient survival were, respectively, 87.5%, 75%, and 69.4% (Figure 1). Univariate analysis did not reveal any factor to impact on overall patient survival (Table 1).

## 4. Discussion

Neoadjuvant therapy has been proven to effectively provide disease control to HCC, avoiding disease progression and enabling liver transplantation within Milan criteria [8]. A recent study has demonstrated that posttransplant outcomes are improved by bridging therapy with TACE [9]. However, selection of neoadjuvant protocols is still based on each center preference, and several different interventions have been proposed to control HCCs, including TACE, TAE, RFA, PEI, and also radiotherapy [10].

Most transplant centers have selected TACE over TAE as main bridging/downstaging therapy for HCC before liver transplant. This policy is reinforced by the EASL Guidelines, which do not recommend TAE as a possible pretransplant therapeutic modality for HCC control during wait-list period [11]. However, there is no strong evidence to support one treatment over the other. A recent study showed no significant differences between wait-list dropout and overall survival between HCC patients undergoing TAE when compared with HCC patients undergoing TACE before liver transplant [5]. Moreover, it also has been demonstrated that TAE was as effective as TACE in reducing HCC recurrence after liver transplant [12].

In the present study, neoadjuvant therapy with TAE, PEI, or both was associated with a 69.4% five-year patient survival. This cohort was comprised only of patients presenting with HCV-related HCC, a subset of HCCs that usually present worse outcomes than HCV negative HCCs [13, 14]. As PEI was employed only for small size (3 cm) HCC nodules, it should only be considered as a bridging but not downstaging therapy. Additionally, 47.5% of the patients had complete HCC necrosis, which is comparable to the rate of complete necrosis found by other studies [12, 15]. Our findings were also in agreement with a recent study employing TACE utilizing a new generation of small beads loaded with doxorubicin [16]. However, as happened to other literature series, complete necrosis was not associated with increased patient survival in our cohort, which may have happened because of the small sample [17].

In our cohort, the effectiveness of TAE and PEI as a neoadjuvant therapies was evaluated without any comparison group. However, our patients achieved long-term survival rates comparable to the best literature outcomes, especially considering that we only included HCV-related HCC patients in the analysis [18, 19]. In this way, TAE associated with PEI followed by liver transplantation enabled a patient survival of 75% at 3 years and 69.4% at 5 years after transplant, results that are at least similar to those achieved with TACE and/or RFA followed by liver transplantation. Moreover, our dropout rate from the wait-list was also low (<10%), reinforcing the argument that TAE is a safe intervention at providing disease control to HCC patients within Milan criteria awaiting for a liver transplant.

Cost-effectiveness is another issue that has acquired importance over the last years. Large referral centers have explored this topic recently, as an attempt to optimize economic resources involved in HCC treatment [20, 21]. However, there is still lack of evidence comparing the cost-effectiveness between distinct neoadjuvant therapeutic options to HCC before liver transplant. In this setting, we have utilized TAE instead of TACE as the first-line therapy for patients with HCC in the wait-list. Since RFA is not available in our public health care system, our protocol also associates PEI for lesions not exceeding 3 cm in diameter whenever technically safe. The estimated cost associated with PEI in our service is under U$ 100 for each therapy session. Each TAE procedure in our hospital costs U$ 200.00 when performed with PVA and U$ 500.00 when microspheres are utilized. It is estimated that TACE would increase the mean costs of TAE by U$ 200 per treatment session (a 40% increase in the costs). Thus, as it has been demonstrated in our cohort, TAE can be used as a safe and cost-effective option to treat HCC before liver transplant.

No variable evaluated here was shown to be associated with decreased survival. This could have been related to the small sample evaluated in this series. Another limitation of the present study is its retrospective design. However, the present study evaluates only HCV-related HCC patients, which increases the homogeneity of the population, potentially strengthening the conclusions.

In conclusion, the use of a bridging therapy to HCV-related HCC that included TAE, PEI, or the association of both TAE and PEI followed by liver transplant not only resulted in an acceptable rate of complete tumor necrosis in the liver explants, but also enabled an excellent 5-year patient survival. This protocol can be effectively used to treat patients with HCC waiting for a liver transplant and can be adopted safely whenever either TACE or RFA is not available option.

## Figures and Tables

**Table 1:** Univariate Cox proportional hazards regression analysis for overall death.

		Hazard ratio (95% CI)	*P* value
Age, years, mean ± standard deviation	58.9 ± 7.4	0.86 (0.08–11.81)	0.91
Gender, female, *N* (%)	17 (42.5)	1.73 (0.57–5.37)	0.33
Under Milan criteria, CT scan, *N* (%)	36 (90)	1.19 (0.23–21.79)	0.86
Under Milan criteria, explant, *N* (%)	22 (55)	1.43 (0.48–4.73)	0.53
Complete HCC necrosis, *N* (%)	19 (47.5)	0.81 (0.26–2.44)	0.71
Single HCC tumor, CT scan, *N* (%)	23 (57.5)	0.65 (0.21–1.95)	0.79
Single HCC tumor, explant, *N* (%)	12 (30)	1.29 (0.35–3.98)	0.67
Vascular invasion, explant, *N* (%)	9 (27.3)^*∗*^	0.54 (0.08–2.1)	0.40
Nuclear grade, undifferentiated, *N* (%)	5 (18.5)^*∗*^	1.03 (0.15–4.4)	0.97

^*∗*^HCCs with complete necrosis were not evaluated for vascular invasion and for nuclear grade.

## Abbreviations

CT: Computed tomography
EAD: Early allograft dysfunction
FFP: Fresh frozen plasma
HCV: Hepatitis C virus
MRI: Magnetic resonance imaging
MELD: Model for End-Stage Liver Disease
PEI: Percutaneous ethanol injection
RBC: Red blood cell unit
TACE: Transarterial chemoembolization
TAE: Transarterial embolization.
